# Changes in Serum TSH and T4 Levels after Switching the Levothyroxine Administration Time from before Breakfast to before Dinner

**DOI:** 10.1155/2015/156375

**Published:** 2015-06-07

**Authors:** S. Ala, O. Akha, Z. Kashi, A. Bahar, H. Askari Rad, N. Sasanpour, A. Shiva

**Affiliations:** ^1^Department of Clinical Pharmacy, Faculty of Pharmacy, Mazandaran University of Medical Sciences, Sari, Mazandaran Province, Iran; ^2^Pharmaceutical Sciences Research Center, Mazandaran University of Medical Sciences, Sari, Mazandaran Province, Iran; ^3^Diabetes Research Center, Mazandaran University of Medical Sciences, Sari, Mazandaran Province, Iran; ^4^Department of Pharmaceutics, Faculty of Pharmacy, Mazandaran University of Medical Sciences, Sari, Mazandaran Province, Iran; ^5^Student Research Committee, Mazandaran University of Medical Sciences, Sari, Mazandaran Province, Iran; ^6^Department of Clinical Pharmacy, Faculty of Pharmacy, Urmia University of Medical Sciences, Urmia, Iran

## Abstract

*Background*. Levothyroxine is commonly used in the treatment of patients with hypothyroidism. Levothyroxine is most often administered in the morning, on an empty stomach, in order to increase its oral absorption. However, many patients have difficulties taking levothyroxine in the morning. *Aim*. The aim of this study was evaluating the effect of changing levothyroxine administration time from before breakfast to before dinner on the serum levels of TSH and T4. *Subjects and Methods*. Fifty patients between 18 and 75 years old with hypothyroidism were included in the study and were randomly divided into two groups. Each group received two tablets per day (one levothyroxine tablet and one placebo tablet) 30 minutes before breakfast and 1 hour before dinner. After two months, the administration time for the tablets was changed for each group, and the new schedule was continued for a further two-month period. The serum TSH and T4 levels were measured before and after treatment in each group. *Results*. Changing the levothyroxine administration time resulted in 1.47 ± 0.51 *µ*IU/mL increase in TSH level (*p* = 0.001) and 0.35 ± 1.05 *µ*g/dL decrease in T4 level (*p* = 0.3). *Conclusions*. Changing the levothyroxine administration time from before breakfast to before dinner reduced the therapeutic efficacy of levothyroxine.

## 1. Introduction

Hypothyroidism is permanent in most patients and requires lifelong thyroid hormone replacement. Replacement with synthetic levothyroxine (LT4) is the mainstay of therapy [1, 2]. Combination therapy with levothyroxine and liothyronine (triiodothyronine or T3) has been suggested as an alternative; however the present evidence from clinical trials does not show any benefit for the combination therapy compared with monotherapy with levothyroxine [3–7]. Recent evidence suggests that the dose of levothyroxine replacement is dependent on sex and body mass but not age as was previously thought [1, 8, 9]. Many factors affect the absorption of levothyroxine; medications such as calcium and iron compounds, aluminium hydroxide, selenium, magnesium, zinc, cholestyramine, sucralfate, raloxifene, proton pump inhibitors, and H2 blockers as well as caffeine, soybean, and fibers can impair absorption of ingested levothyroxine [1, 2, 10–12]. Phenytoin, carbamazepine, phenobarbital, and rifampicin can increase the clearance of levothyroxine [2]. Thus, it should be taken on an empty stomach, without other medications, supplements, or food for 1 hour or in a similar fashion 4 hours after the last meal. A fasting regimen of administration helps to ensure that the TSH remains within a narrow target range [1, 2]. The usual time schedule for taking levothyroxine tablets in patients with hypothyroidism is every morning, before breakfast. However, some patients have difficulties taking their medication at early morning due to gastrointestinal disturbance. Thus, several studies have been performed on the efficacy of evening doses of levothyroxine [13–16]. However, the literature data are inconsistent and contradictory. It has been demonstrated in studies by Bartalena et al. [13] and Bolk et al. [14, 15] that changing the levothyroxine administration time from morning (before breakfast) to bedtime (after dinner) leads to increased absorption and increased efficacy of levothyroxine (as evident from reduced levels of TSH). On the other hand, Bach-Huynh et al. [16] reported increased serum TSH levels and reduction in serum T4 levels in response to changing the levothyroxine administration time from morning to evening. A more recent study by Rajput et al. [17] demonstrated equal efficacy for morning and evening doses of levothyroxine.

The objective of this study was investigating the effect of changing the levothyroxine administration time from before breakfast to before dinner on serum TSH land T4 levels in patients with primary hypothyroidism. This administration schedule was opted for in order to evade the possibility of taking the levothyroxine tablets on a full stomach as a result of short interval between dinner time and bedtime (according to the general trait in the region where the study was conducted) and to reduce the possibility of forgetting the bedtime doses.

## 2. Subjects and Methods

### 2.1. Trial Design

The present study was a prospective, randomized, double-blind, crossover placebo controlled study. The study was approved by the Medical Research Ethics Committee of Mazandaran University of Medical Sciences and registered at Iranian Registry of Clinical Trials with registration number IRCT138903223014N2 (the full trial protocol could be accessed online at http://www.irct.ir/).

### 2.2. Patient Selection

Patients between 18 and 75 years, of both sexes, with hypothyroidism (based on the physicians diagnosis), referred to Tuba Medical Center, Sari, Iran, were considered for inclusion in the study. Informed written consent was obtained from all of the patients prior to enrollment in the study. Patients with a history of gastrointestinal disorders, chronic pulmonary disorders, chronic cardiovascular diseases, renal failure, diabetes, concomitant use of medications that interfere with absorption or metabolism of levothyroxine (such as cholestyramine and antibiotics), and pregnant women were excluded from the study. To ensure the normal levels of TSH and T4, all of the patients underwent 3 laboratory tests (with 15-day intervals) before commencement of the study. In cases of TSH and T4 values above or below the normal range, the patients were subjected to levothyroxine dose adjustment and followed up until normal serum levels of TSH and T4 were achieved.

### 2.3. Trial Procedure

The patients were randomly divided into two groups following simple randomization procedure using a computer generated list of random numbers. Patients of both groups received one batch of levothyroxine and one batch of placebo and were recommended to take the tablets with a 12-hour interval (one before breakfast and one before dinner), with the predetermined dosage. The levothyroxine tablets and the placebo tablets were prepared by the same manufacturer (Iran Hormone Co., Tehran, Iran), were identical in shape, color, and size, and were packed in similar blisters. The blisters were coded with labels of different colors (yellow in the case of placebo and green in the case of levothyroxine). Neither the patients nor the physicians were aware of the randomization codes until the end of the study.

The study consisted of two 60-day courses. During the first course, group A received the levothyroxine tablets in the morning, 30 minutes before breakfast, and the placebo tablet 1 hour before dinner, whereas group B received the levothyroxine and placebo tablets in reverse order. During the second course, the levothyroxine and placebo administration times were switched within each group. The primary outcomes were the serum levels of T4 and TSH which were measured at the end of the first and second course (at the 60th and 120th day of the study), by ELISA (enzyme linked immunosorbent assay) technique. Blood samples were drawn between 8 AM and 9 AM. The serum concentration values of 0.39–6.1 *μ*IU/mL for TSH and 4.8–11.6 *μ*g/dL for T4 were considered to be normal.

### 2.4. Statistical Analysis

The statistical analysis of the data was carried out by SPSS software version 16. The paired sample *t*-test was used to compare the data and values of *p* less than 0.05 were considered to denote a significant difference in all cases.

## 3. Results

**Figure 1:**
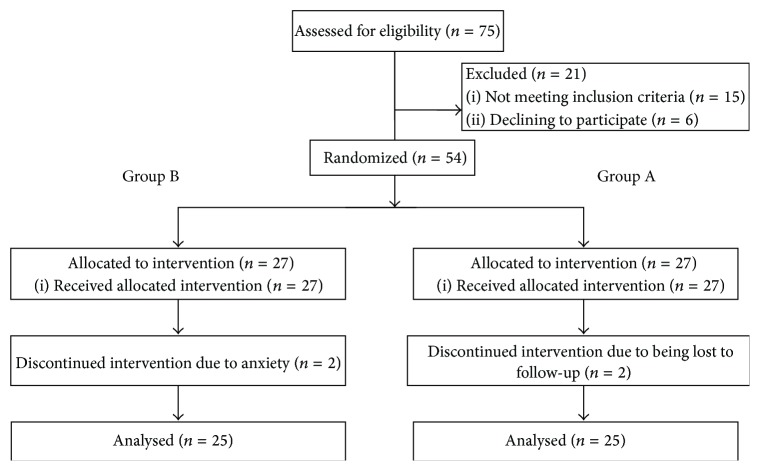
Participant flow diagram (according to guidelines of CONSORT 2010).

A total number of 54 patients between 18 and 67 years were recruited in the study. Of these, two patients (one in each arm) discontinued the study due to fear that changing the therapeutic regimen might deteriorate their disease and two patients (one in each arm) were lost to follow-up leaving 50 patients for analysis (25 patients in each group). The full participant flow diagram is depicted in Figure 1. The average administered dose of levothyroxine in the whole study population was 0.1 mg/day.

The study population was rather young as the majority of patients (33 or 66%) were less than 40 years (Table 1).

In both groups the within group differences in the serum TSH and T4 levels as a result of changing the administration time were similar. There was significant increase in average TSH level (*p*_1_ = 0.04, *p*_2_ = 0.035 for group A and group B, resp.), whereas the decrease in average T4 level was insignificant (*p*_1_ = 0.7, *p*_2_ = 0.64 for group A and group B, resp.). Since the results in both crossed over groups was the same, the two groups could be regarded as one.

From the 50 patients included in the study, in 38 patients (76%) changing the levothyroxine administration time from morning to evening increased the serum levels of TSH significantly (*p* < 0.05), and in the remaining 12 patients (24%), the TSH levels decreased or remained constant; in regard to T4, in 33 patients (66%) the serum levels of T4 decreased, whereas in the remaining 17 patients (34%), the serum levels of T4 increased as a result of changing the levothyroxine administration time from morning to evening; however, the difference was not significant (*p* > 0.05). The average values of TSH and T4 at different points during the trial are depicted in Tables 2 and 3. To investigate the effect of age on the dependant variables, the data were evaluated in regard to different age groups (less than 40 years and more than 40 years). There were not any significant differences in the values of TSH or T4 between the two age groups when levothyroxine was administered before breakfast (*p*_1_ = 0.051 and *p*_2_ = 0.6, resp.) or when levothyroxine was administered before dinner (*p*_1_ = 0.3 and *p*_2_ = 0.1, resp.). Thus, it seems that the patients' age did not influence the therapeutic outcome.

## 4. Discussion

The current therapeutic procedure for hypothyroidism is mainly focused on hormone replacement therapy by sodium levothyroxine. The patients are usually advised to take the medication in the morning 30–60 minutes before breakfast. However, for many patients, this time schedule is not appropriate and they feel more comfortable to take the medication in the evening. In this study, the effect of changing levothyroxine administration time from morning to evening on the serum levels of TSH and T4 was evaluated. The effects of changing the levothyroxine administration time on serum TSH and T4 levels were previously studied [13–17], but the literature data were inconsistent and contradictory.

Bartalena et al. demonstrated that greatest variations in serum concentrations of TSH are obtained when levothyroxine is administrated either early in the morning or late in the evening [13]. Bolk et al. studied the effects of levothyroxine administration time (morning versus evening) on the serum levels of TSH and T4 in 12 female patients for 4 months and found that administration of levothyroxine in the evening results in decreased serum levels of TSH [14]. However, due to the small sample size in this study, the results were not considered to be generalizable. Thus, in a more extended study by Bolk et al. later on, 105 patients were studied for a period of 6 months, with a shift in levothyroxine administration time from morning to evening and the results showed greater absorption for levothyroxine, decreased serum levels of TSH, and increased levels of T4 when levothyroxine was administered at bed time [15]. Bach-Huynh et al. conducted a similar study including 105 patients for 24 weeks. Their study, in contrast, demonstrated an increase in the serum TSH levels and reduction in serum T4 levels in response to changing the levothyroxine administration time from morning to evening [16]. In a more recent study, Rajput et al. studied 152 drug naïve patients with primary hypothyroidism for the effects of morning versus evening administration of levothyroxine on the clinical profile and quality of life. The patients were divided into two groups receiving levothyroxine either in the morning or in the evening on an empty stomach for a period of 12 weeks. The results demonstrated considerable improvement in clinical profile for majority of patients in both groups with no significant between group differences, and the evening administration of levothyroxine was reported to be as efficacious as the morning administration [17].

In the present study, changing the administration time of levothyroxine from before breakfast to before dinner resulted in a considerable increase in the serum levels of TSH, in the entire study population (*p* = 0.001). However, the changes in serum T4 levels were insignificant and negligible (*p* = 0.3). This is in accordance with the results reported by Bach-Huynh et al. [16], who demonstrated a 1.25 *μ*IU/mL increase in average serum level of TSH as a result of changing the levothyroxine administration time from morning to evening. One possible proposed mechanism is that dinner content may have more impact on levothyroxine absorption versus breakfast, even if eaten an hour after ingestion of the tablet.

Rajput et al. [17] found equal efficacy of the two administration times but this contradicted the results obtained by Bolk et al. [14, 15]. This inconsistency might be in part due to the nutrition regimen in different patients and the effects of food intake on the absorption and oral bioavailability of levothyroxine. Data from screening large European population have revealed the influence of dietary iodine intake on the epidemiology of thyroid dysfunction [18].

Comparison of the TSH and T4 levels between the age groups less than 40 years and more than 40 years did not demonstrate any significant difference either in initial or in final levels of TSH and T4. In addition there was no relationship between the BMI and the age group. Although the absorption, distribution, metabolism, and excretion of levothyroxine, like any other drug, depend on age and BMI of the patient, since the daily dose of levothyroxine was precisely determined for each patient by the endocrinologist physician, on the basis of the preliminary conditions and the extent of hypothyroidism, these factors did not affect the study variables in different age groups.

The study population was predominantly composed of women (88%), reflecting the fact that the prevalence of hypothyroidism is higher in women compared with men. This was also reported by Assadi et al. [19] who studied 2000 Iranian patients aged above 20 years for subclinical thyroid disorders. Their study demonstrated higher prevalence of thyroid disorders in women compared with men (60.7% versus 39.3% of the study population, resp.) and higher prevalence of hypothyroidism for women compared with men (11.87 per 10000 versus 4.9 per 1000).

The main limitations of this study include single-centre site and the inability to monitor patients' compliance and achieving a stable dose of levothyroxine was not anticipated as inclusion criterion for this study.

Although the serum levels of T4 were not significantly changed, the changes to serum TSH levels were significant, suggesting changing the levothyroxine administration time from before breakfast to before dinner in order to enhance the patient compliance results in reduced therapeutic outcome. Also comparing our results with previous studies suggests that taking levothyroxine before bedtime, that is, after food, could have led to better bioavailability than the predinner administration used in this study.

## Figures and Tables

**Table 1:** Demographic characteristics of the patients (*n* = 50).

Characteristic	Value
Female/male ratio	44/6
Mean age (years)	37 ± 13.2
Age groups	
18–30 years	18 (36%)
30–40 years	15 (30%)
>40 years	17 (34%)
Body mass index	
19–25	18 (36%)
25–30	18 (36%)
>30	14 (28%)
Familial history of hypothyroidism	
Yes	10 (20%)
No	40 (80%)
Etiology of hypothyroidism	
Autoimmune disease	41 (82%)
Thyroidectomy	5 (10%)
Radiation therapy	4 (8%)
Concurrent disease	
None	39 (78%)
Iron deficiency anemia	5 (10%)
Hyperlipidemia	2 (4%)
Hypertension and hyperlipidemia	4 (8%)

**Table 2:** Changes to the serum levels of TSH during the study (all data are reported as mean ± SD).

Age group	Serum TSH (*μ*IU/mL)			
	BB^1^	BD^2^	Difference	*p* value
≤40 years (*n* = 33)	2.26 ± 1.19	3.52 ± 1.59	1.26 ± 0.4	0.00
>40 years (*n* = 17)	1.55 ± 1.14	2.99 ± 1.98	1.44 ± 0.84	0.02
Total population (*n* = 50)	2.03 ± 1.22	3.35 ± 1.73	1.47 ± 0.51	0.00

^1^Before breakfast, ^2^before dinner.

**Table 3:** Changes to the serum levels of T4 during the study (all data are reported as mean ± SD).

Age group	Serum T4 (*μ*g/dL)			
	BB^1^	BD^2^	Difference	*p* value
≤40 years (*n* = 33)	8.87 ± 2.62	8.42 ± 1.25	0.45 ± 1.37	0.4
>40 years (*n* = 17)	9.20 ± 1.62	9.05 ± 1.25	0.15 ± 0.37	0.7
Total population (*n* = 50)	8.98 ± 2.32	8.63 ± 1.27	0.35 ± 1.05	0.3

^1^Before breakfast, ^2^before dinner.
